# Supplementary material for: A Bivalent Molecular Glue Linking Lysine Acetyltransferases to Oncogene-induced Cell Death
Source: bioRxiv. 2025 Mar 17:2025.03.14.643404. Preprint. [Version 1] doi: 10.1101/2025.03.14.643404 (PMC11956963; doi:10.1101/2025.03.14.643404)

A

Full scans for Supplemental Fig. 4F

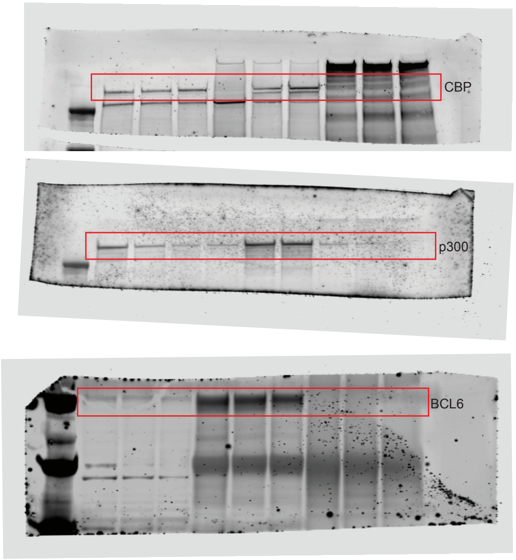

B

Full scans for Fig. 3A

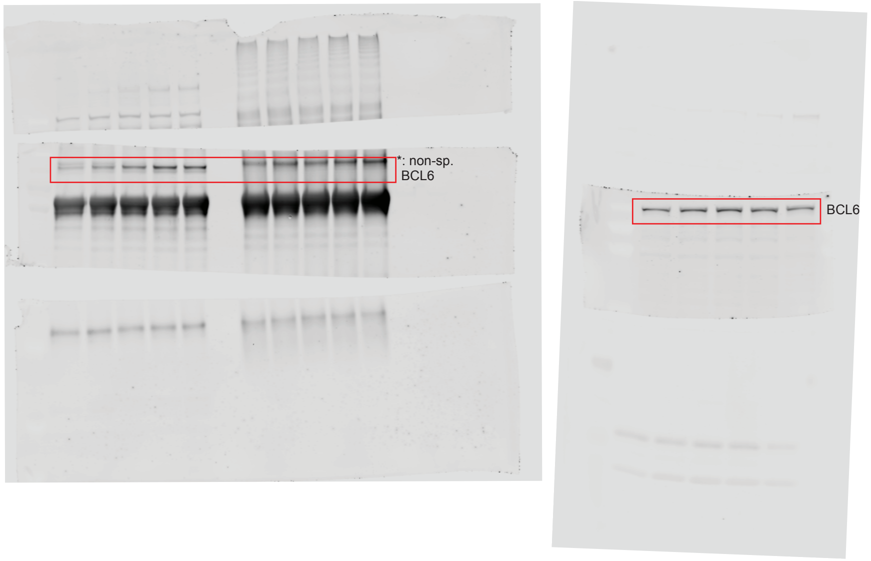

C

Full scans for Fig. 3G

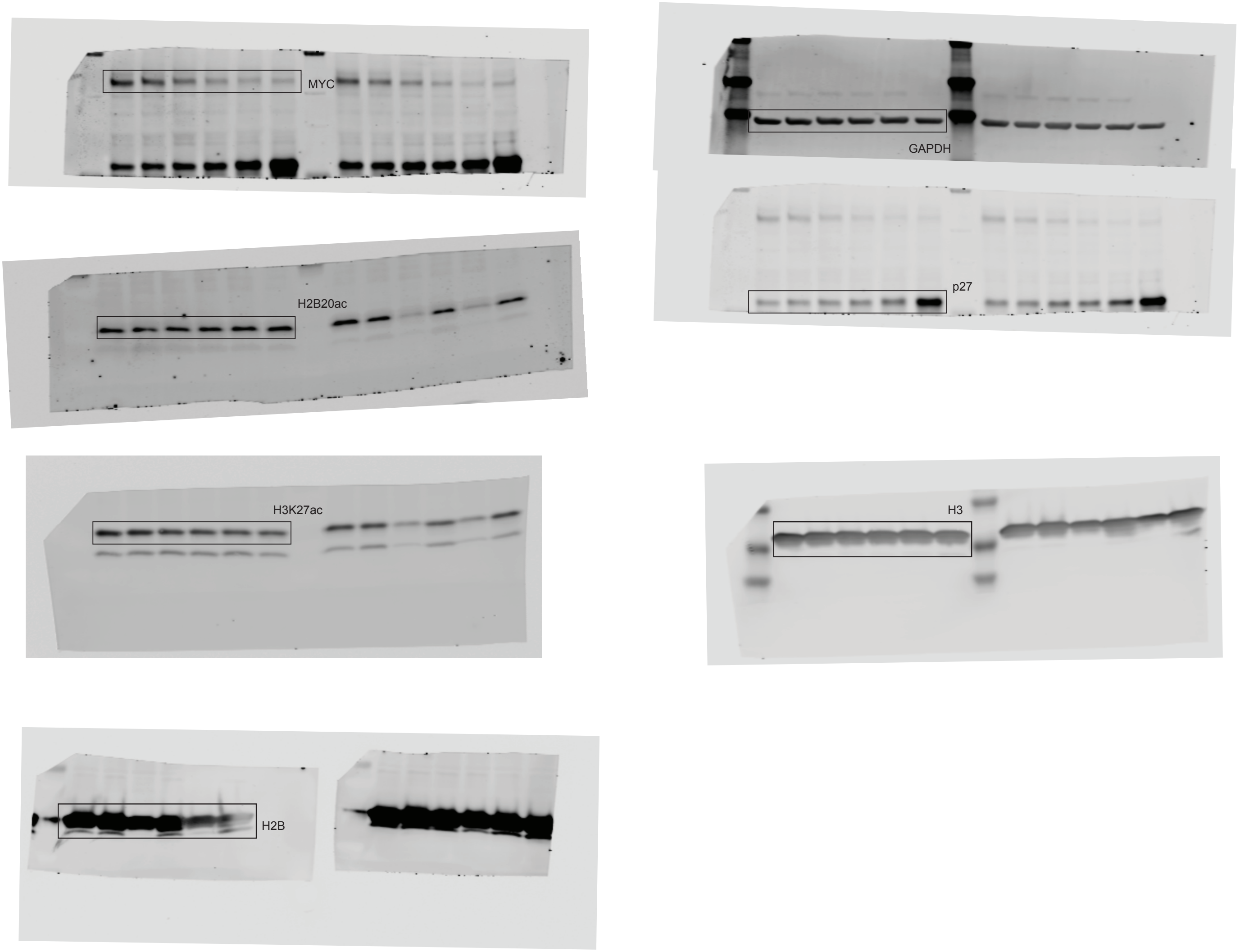

D

Full scans for Fig. 4D

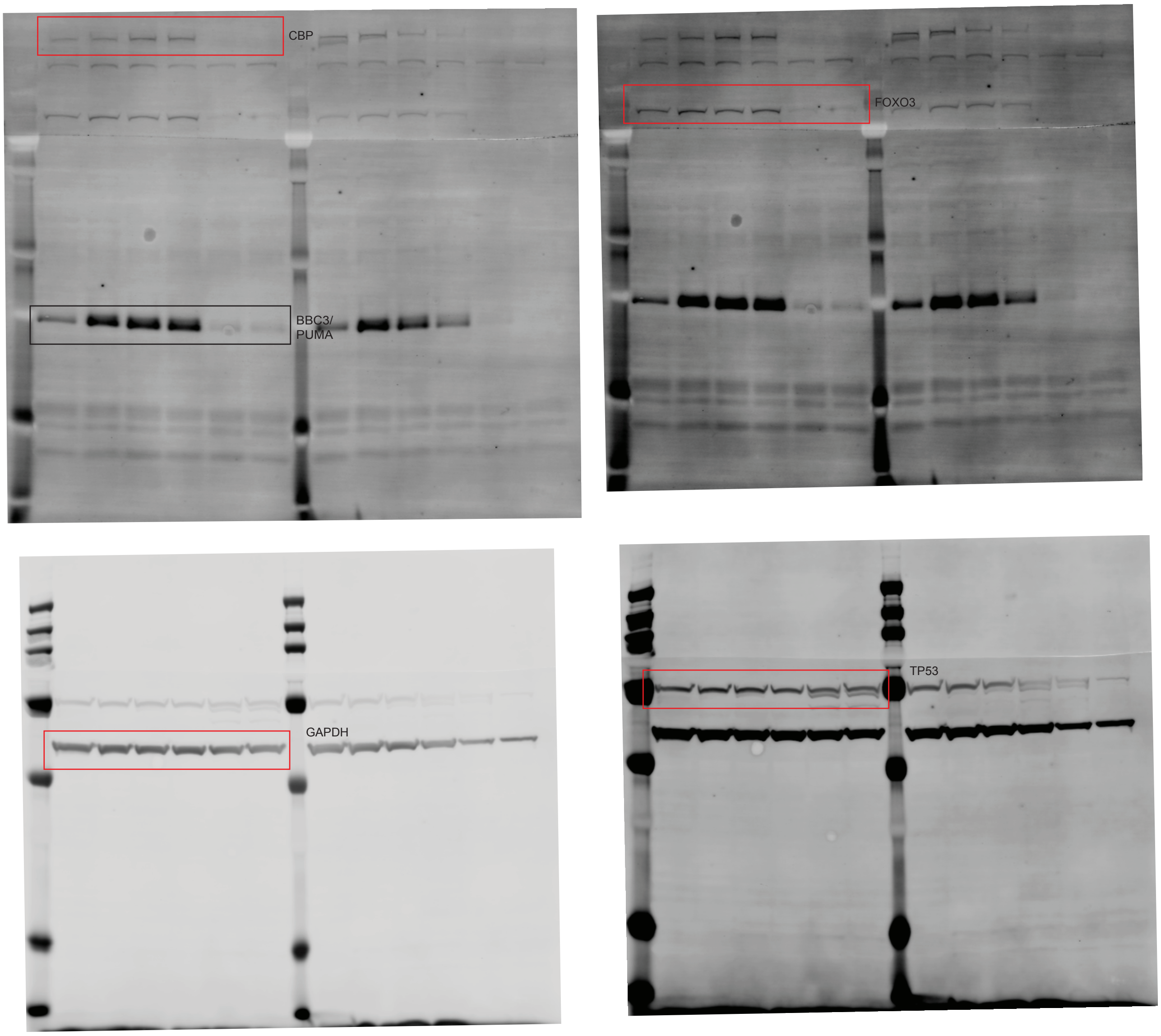

E

Full scans for Supplemental Fig. 7C

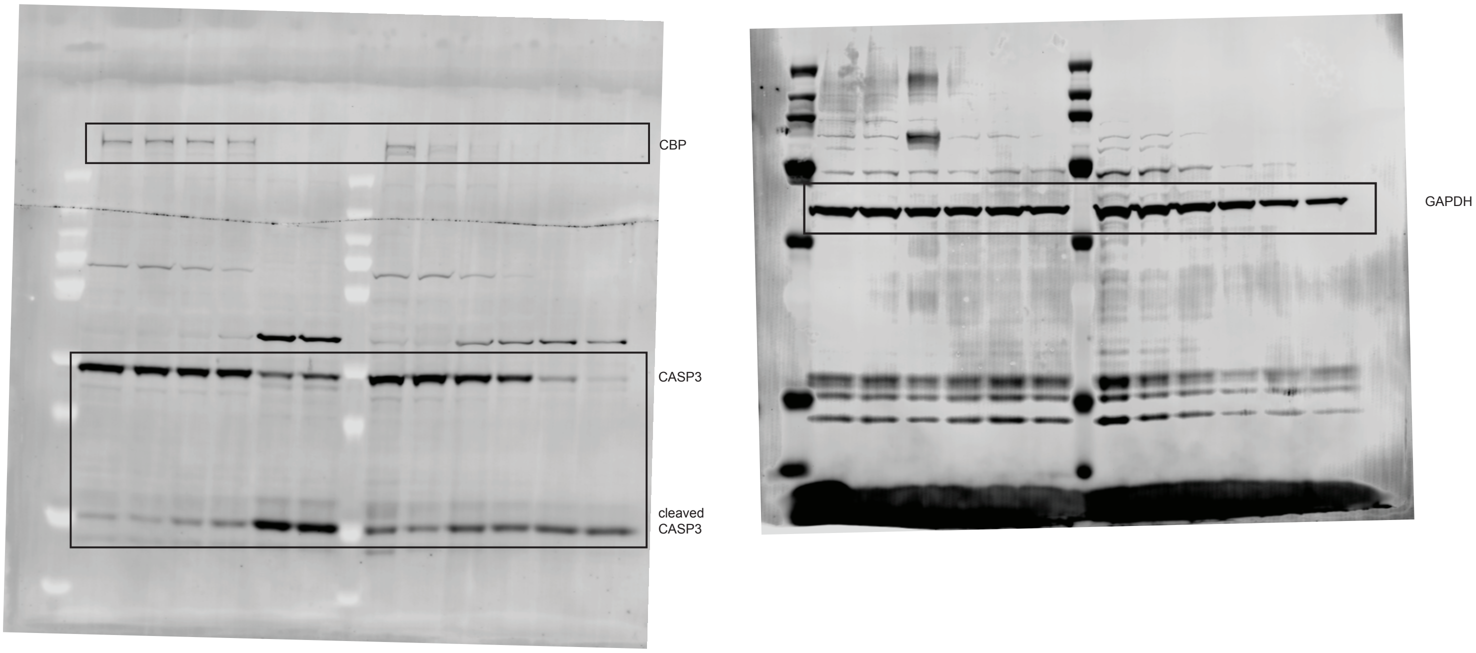

F

Full scans for Fig. 5C

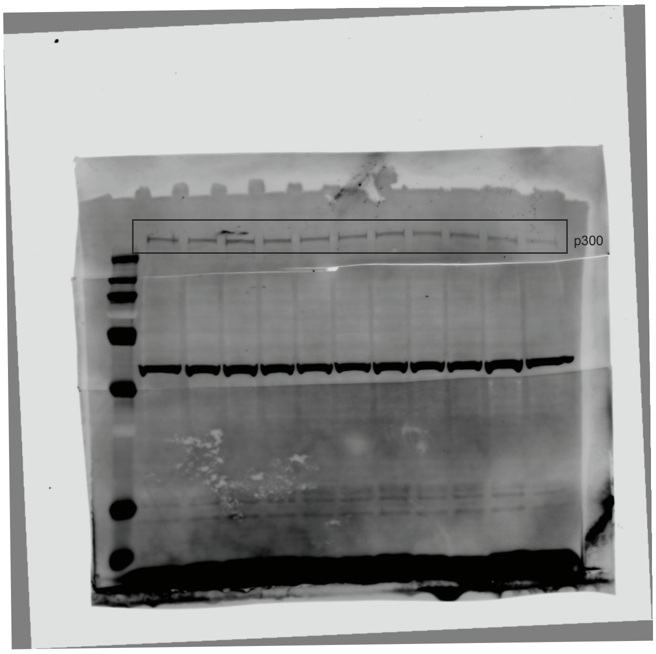

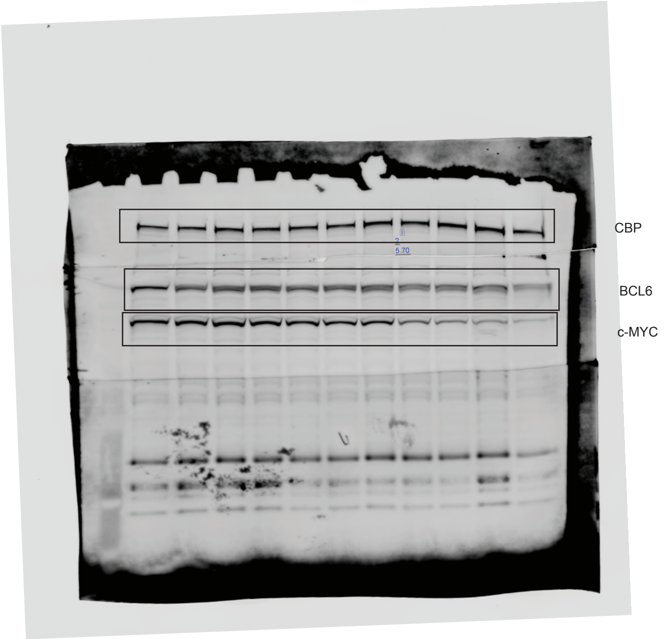

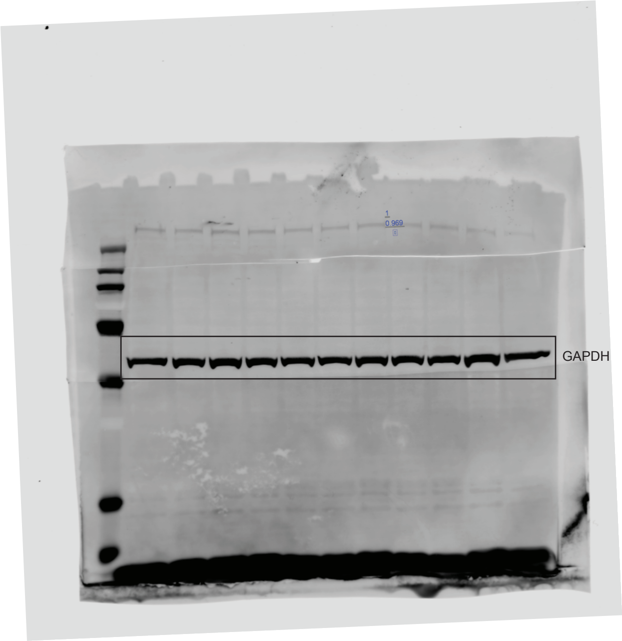

G

Full scans for Fig. 5I

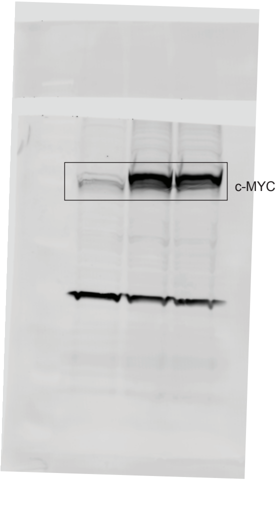

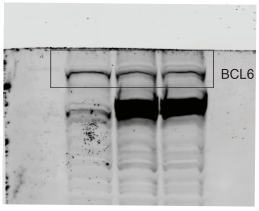

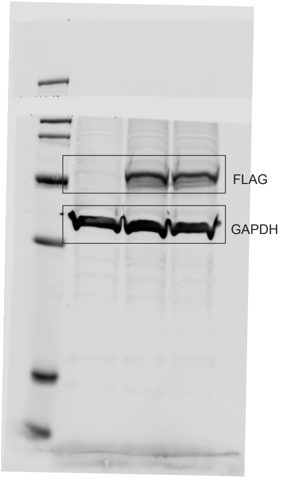

H

Full scans for Supplemental Fig. 9A

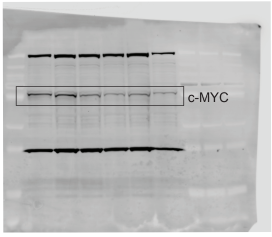

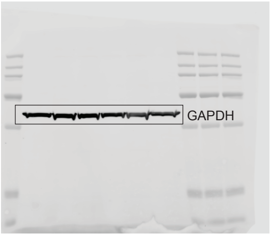

I

Full scans for Supplemental Fig. 9B

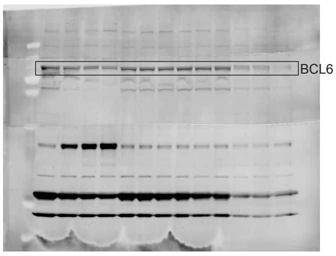

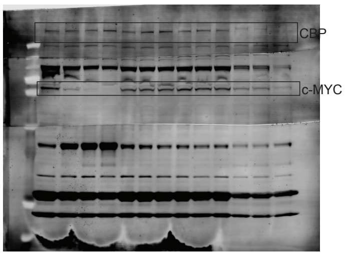

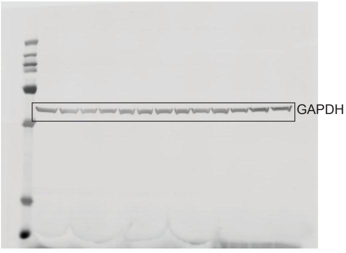

Supplement: Supplement 2 [file media-2.pdf]
