# Supplementary material for: A Bivalent Molecular Glue Linking Lysine Acetyltransferases to Oncogene-induced Cell Death
Source: bioRxiv. 2025 Mar 17:2025.03.14.643404. Preprint. [Version 1] doi: 10.1101/2025.03.14.643404 (PMC11956963; doi:10.1101/2025.03.14.643404)
Supplement: Supplement 3 [file media-3.pdf]

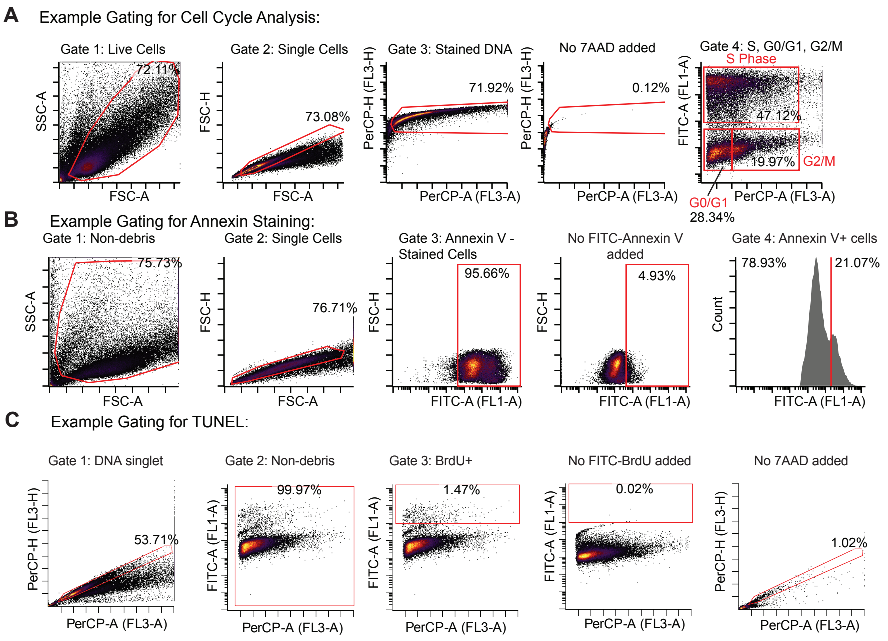
A
Example Gating for Cell Cycle Analysis:
Gate 1: Live Cells
72.11%
SSC-A
FSC-A
Gate 2: Single Cells
73.08%
FSC-H
FSC-A
Gate 3: Stained DNA
71.92%
PerCP-H (FL3-H)
PerCP-A (FL3-A)
No 7AAD added
0.12%
PerCP-H (FL3-H)
PerCP-A (FL3-A)
Gate 4: S, G0/G1, G2/M
S Phase
47.12%
19.97%
G2/M
G0/G1
28.34%
FITC-A (FL1-A)
PerCP-A (FL3-A)
B
Example Gating for Annexin Staining:
Gate 1: Non-debris
75.73%
SSC-A
FSC-A
Gate 2: Single Cells
76.71%
FSC-H
FSC-A
Gate 3: Annexin V - Stained Cells
95.66%
FSC-H
FITC-A (FL1-A)
No FITC-Annexin V added
4.93%
FSC-H
FITC-A (FL1-A)
Gate 4: Annexin V+ cells
78.93%
21.07%
Count
FITC-A (FL1-A)
C
Example Gating for TUNEL:
Gate 1: DNA singlet
53.71%
PerCP-H (FL3-H)
PerCP-A (FL3-A)
Gate 2: Non-debris
99.97%
FITC-A (FL1-A)
PerCP-A (FL3-A)
Gate 3: BrdU+
1.47%
FITC-A (FL1-A)
PerCP-A (FL3-A)
No FITC-BrdU added
0.02%
FITC-A (FL1-A)
PerCP-A (FL3-A)
No 7AAD added
1.02%
PerCP-H (FL3-H)
PerCP-A (FL3-A)
