# Supplementary material for: A Bivalent Molecular Glue Linking Lysine Acetyltransferases to Oncogene-induced Cell Death
Source: bioRxiv. 2025 Mar 17:2025.03.14.643404. Preprint. [Version 1] doi: 10.1101/2025.03.14.643404 (PMC11956963; doi:10.1101/2025.03.14.643404)
Supplement: 1 [file NIHPP2025.03.14.643404V1-supplement-1.pdf]

bioRxiv preprint doi: https://doi.org/10.1101/2025.03.14.643404; this version posted March 17, 2025. The copyright holder for this preprint (which was not certified by peer review) is the author/funder, who has granted bioRxiv a license to display the preprint in perpetuity. It is made available under aCC-BY 4.0 International license.

SUPPLEMENTAL FIGURES

bioRxiv preprint doi: https://doi.org/10.1101/2025.03.14.643404; this version posted March 17, 2025. The copyright holder for this preprint (which was not certified by peer review) is the author/funder, who has granted bioRxiv a license to display the preprint in perpetuity. It is made available under aCC-BY 4.0 International license.

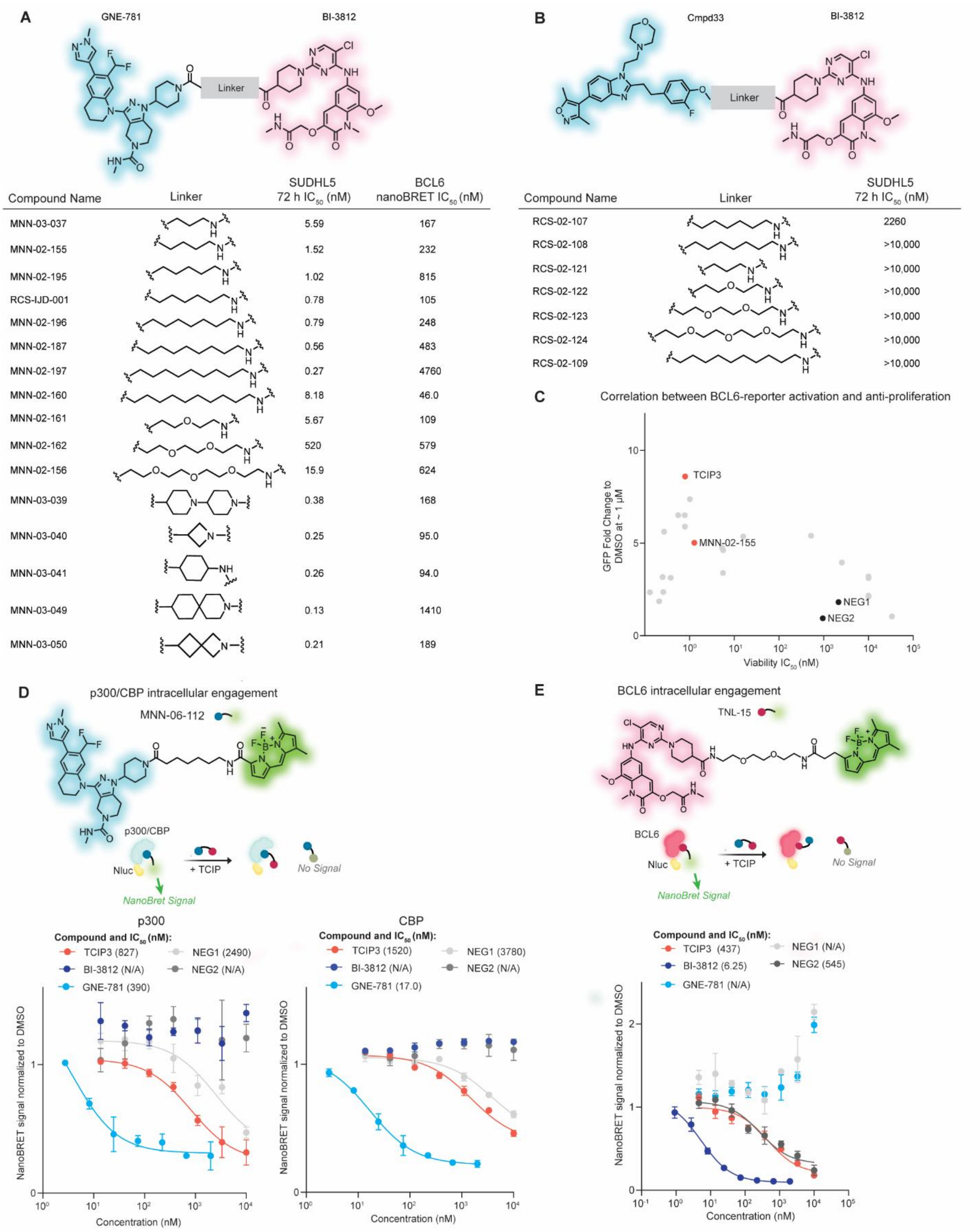

Supplemental Figure 1. Structure-Activity Relationship of p300/CBP KAT-TCIP Library

(A) Design of GNE-781 and **(B)** Cmpd33-based KAT-TCIP libraries and corresponding IC_{50} values of cell viability after 72 h treatment in SUDHL5 and $BCL6^{BTB}$ intracellular

bioRxiv preprint doi: https://doi.org/10.1101/2025.03.14.643404; this version posted March 17, 2025. The copyright holder for this preprint (which was not certified by peer review) is the author/funder, who has granted bioRxiv a license to display the preprint in perpetuity. It is made available under aCC-BY 4.0 International license.

probe-displacement (nanoBRET: nano-bioluminescence resonance energy transfer) in HEK293T cells; for cell viability, mean of 1-4 biological replicates; for nanoBRET, mean of 3 technical replicates.

(C) Reporter transactivation (fold change of BCL6-repressed GFP) after 24 h of treatment in K422 reporter cells versus IC_{50} values of cell viability after 72 h treatment in SUDHL5 for all KAT-TCIP compounds; for cell viability, mean of 1-4 biological replicates.

(D) Assessment of compounds and corresponding IC_{50} values of p300 and CBP intracellular probe-displacement in 293T cells; the probe **MNN-06-112** is shown; Nluc: nano-luciferase.

(E) Assessment of compounds and corresponding IC_{50} values in BCL6 nanoBRET in HEK293T cells; the probe **TNL-15** is shown. For **(D)**, **(E)**, mean ± s.e.m of 3 technical replicates.

bioRxiv preprint doi: https://doi.org/10.1101/2025.03.14.643404; this version posted March 17, 2025. The copyright holder for this preprint (which was not certified by peer review) is the author/funder, who has granted bioRxiv a license to display the preprint in perpetuity. It is made available under aCC-BY 4.0 International license.

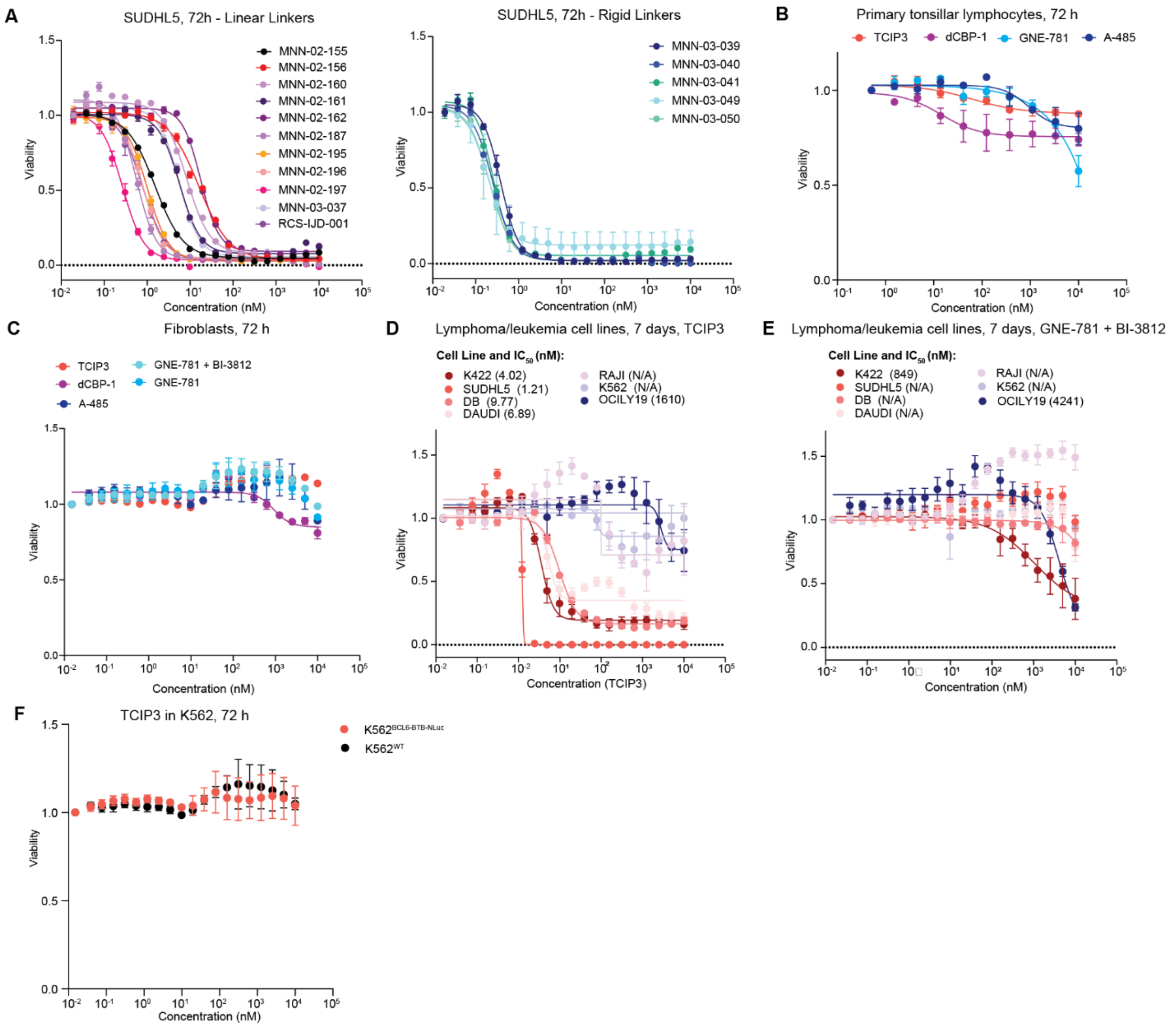

Supplemental Figure 2. Assessment of KAT-TCIP Toxicity in Primary and DLBCL Cells

(A) Cell viability curves of GNE-781-based KAT-TCIPs containing either linear or rigid linkers after 72 h treatment in SUDHL5 cells; corresponds to summary data in Supplemental Fig. 1A; mean ± s.e.m. of 2-3 biological replicates or 3 technical replicates.

(B) Viability effects of **TCIP3** and known p300/CBP targeting agents in primary tonsillar lymphocytes from two independent donors (Methods) after treatment for 72 h; mean ± s.e.m.

(C) Viability effects of **TCIP3** and known p300/CBP targeting agents in human fibroblasts after treatment for 72 h; 2 biological replicates; mean ± s.e.m.

(D) Viability effects of 7 days of **TCIP3** treatment in DLBCL and leukemia cell lines; 3-4 biological replicates; mean ± s.e.m.

(E) Viability effects of 7 days of BI-3812 and GNE-781 co-treatment in DLBCL and leukemia cell lines; 3-4 biological replicates; mean ± s.e.m.

(F) Viability effects after 72 h of **TCIP3** treatment in K562 cells overexpressing NLuc-BCL6^{BTB}; 3 biological replicates; mean ± s.e.m.

bioRxiv preprint doi: https://doi.org/10.1101/2025.03.14.643404; this version posted March 17, 2025. The copyright holder for this preprint (which was not certified by peer review) is the author/funder, who has granted bioRxiv a license to display the preprint in perpetuity. It is made available under aCC-BY 4.0 International license.

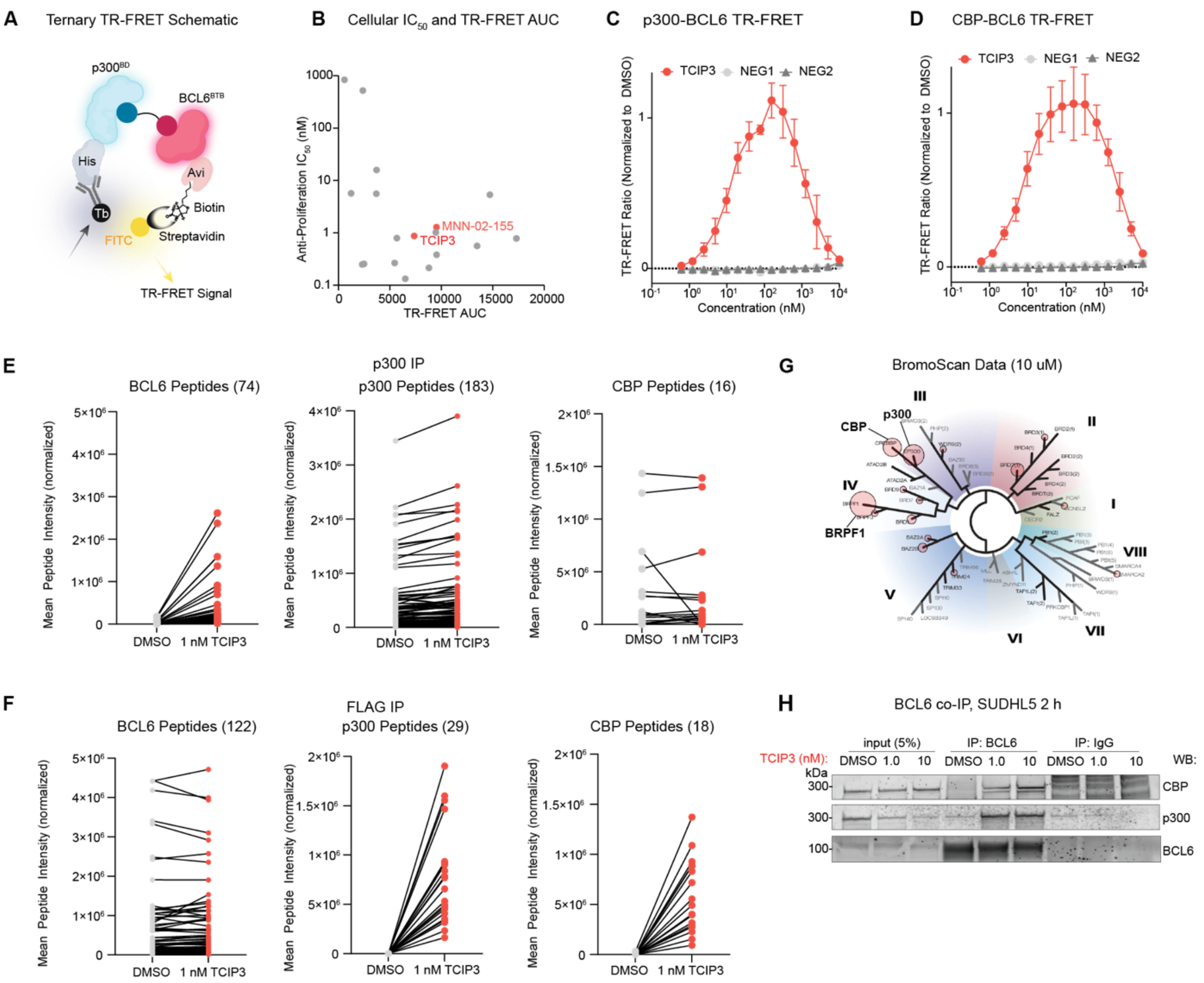

Supplemental Figure 3. Biochemical and Cellular Engagement of BCL6 and p300/CBP

(A) Design of ternary TR-FRET assay using recombinant 6x-His-p300^{BD} or 6x-His-CBP^{BD} and biotinylated BCL6^{BTB}-Avi to measure ternary complex formation.

(B) Antiproliferation IC_{50} (nM) vs TR-FRET curve (AUC) for KAT-TCIPs synthesized from GNE-781; for TR-FRET, mean of 3 biological replicates each with 3 technical replicates; for cell viability IC_{50}s, mean of 1-4 biological replicates.

(C) p300-BCL6 or **(D)** CBP-BCL6 TR-FRET assay of **TCIP3, NEG1,** or **NEG2**; mean ± s.e.m. of 3 technical replicates.

(E) Mean peptide intensities of BCL6, p300, or CBP matched between DMSO or **TCIP3** treatments in SUDHL5 cells treated for 2 h for p300 IP-MS shown in Figure 2C.

(F) Mean peptide intensities of BCL6, p300, or CBP matched between DMSO or **TCIP3** treatments in FLAG-tagged *BCL6* SUDHL5 cells treated for 2 h for FLAG IP-MS shown in Figure 2D. For **E** and **F**, total number of unique, proteotypic peptides across all conditions provided in heading parentheses.

(G) Selectivity of binding to 40 recombinant human bromodomains (BROMOscan) with **TCIP3** (10 μM).

(H) BCL6 IP from SUDHL5 cells treated with the indicated doses of **TCIP3** for 2 h; representative of 3 biological replicates.

bioRxiv preprint doi: https://doi.org/10.1101/2025.03.14.643404; this version posted March 17, 2025. The copyright holder for this preprint (which was not certified by peer review) is the author/funder, who has granted bioRxiv a license to display the preprint in perpetuity. It is made available under aCC-BY 4.0 International license.

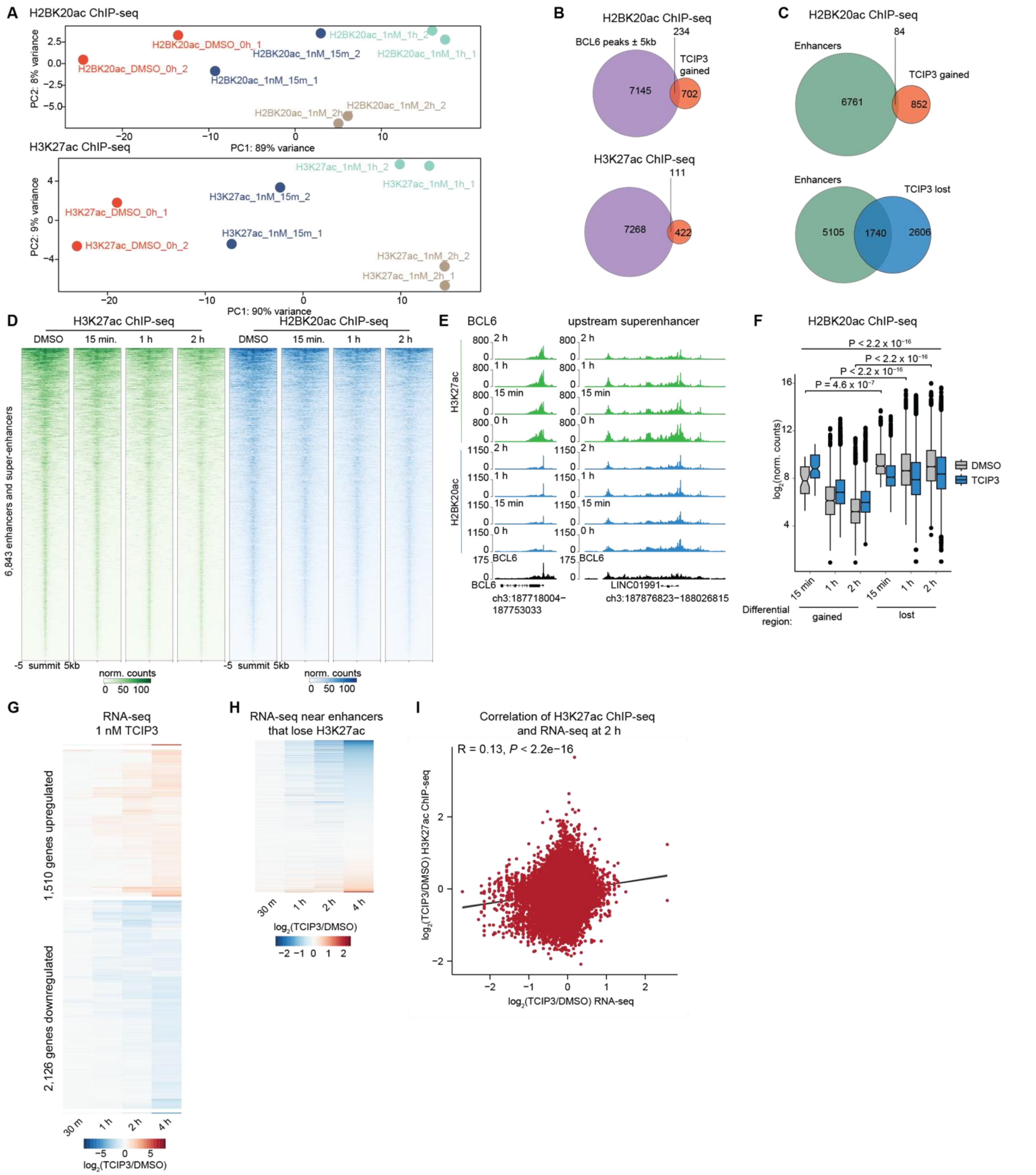

Supplemental Figure 4. Reprogramming of Histone Lysine Acetylation and Gene Expression

(A) Principal component analysis of biological replicates for H3K27ac (2 per timepoint) and H2BK20ac (2 per timepoint) ChIP-seq experiments.

(B) Overlap of gained H2BK20ac and H3K27ac peaks after 1 h of 1 nM TCIP3 with BCL6 summits ± 5 kilobases (kb) in SUDHL5 cells as measured by BCL6 CUT&RUN.

bioRxiv preprint doi: https://doi.org/10.1101/2025.03.14.643404; this version posted March 17, 2025. The copyright holder for this preprint (which was not certified by peer review) is the author/funder, who has granted bioRxiv a license to display the preprint in perpetuity. It is made available under aCC-BY 4.0 International license.

(C) Overlap of gained and lost H2BK20ac peaks after 1 h of 1 nM **TCIP3** with annotated enhancers and super-enhancers in SUDHL5 cells.; differential regions defined as in **Fig. 3A**.
(D) H3K27ac and H2BK20ac at all enhancers and super-enhancers for the indicated timepoints of **TCIP3** treatment; merged from 2 biological replicates and sequence-depth normalized and input-subtracted.
(E) Induction of H2BK20ac and H3K27ac with time at the promoter of *BCL6* concomitant with loss at the *BCL6* upstream super-enhancer; BCL6 track is CUT&RUN in untreated SUDHL5 cells, tracks merged from two biological replicates and sequence-depth normalized and, for histone acetylation ChIP-seq, also input-subtracted.
(F) Comparison of H2BK20ac loading at differential regions at 15 min, 1 h, and 2 h of 1 nM **TCIP3**; *P*-values adjusted by Tukey's test after type II analysis of variance (ANOVA).
(G) Time-dependent changes in gene expression; plotted are differential genes defined by adj. $P \leq 0.05$ and $|\log_2(\text{fold change})| \geq 0.5$, *P*-values computed by two-sided Wald test and adjusted for multiple comparisons by Benjamini-Hochberg.
(H) Changes in gene expression at genes near enhancers and super-enhancers that had statistically significant (adj. $P \leq 0.05$ and $\log_2(\text{fold change}) \leq -0.5$) losses in H3K27ac as measured by ChIP-seq **Fig. 3E**.
(I) Correlation of changes at all H3K27ac peaks (66,995) after 2 h of 1 nM **TCIP3** treatment with changes in gene expression of nearest gene to peak; *R* computed by Pearson's correlation and *P*-value computed by two-sided Student's t-test.

bioRxiv preprint doi: https://doi.org/10.1101/2025.03.14.643404; this version posted March 17, 2025. The copyright holder for this preprint (which was not certified by peer review) is the author/funder, who has granted bioRxiv a license to display the preprint in perpetuity. It is made available under aCC-BY 4.0 International license.

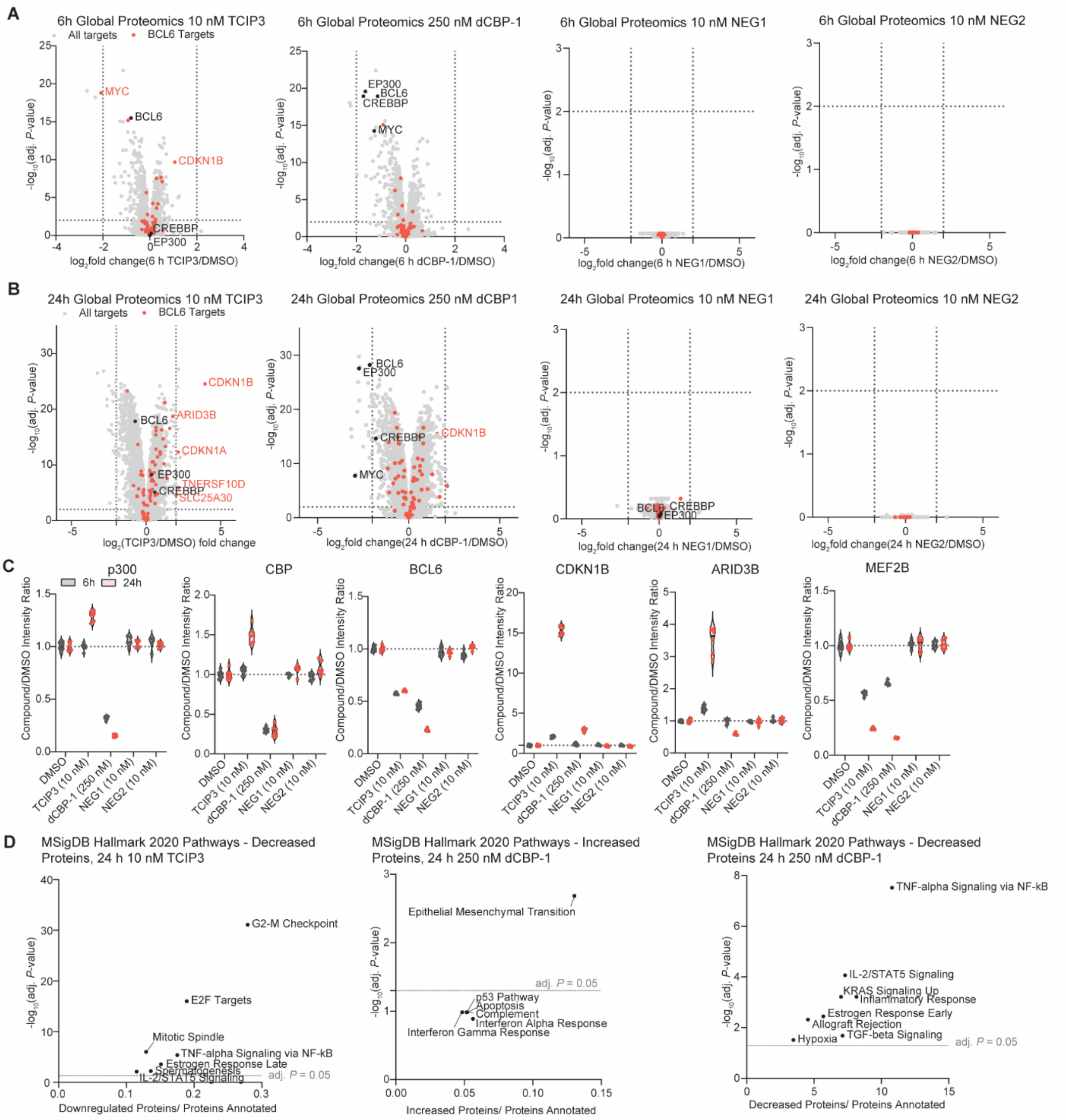

Supplemental Figure 5. Proteomic Effects of TCIP3, dCBP1, NEG1, and NEG2

(A) Whole-proteome profiling of SUDHL5 cells treated with10 nM **TCIP3**, 250 nM dCBP1, 10 nM **NEG1**, or 10 nM **NEG2** for 24 h plotted with cutoffs of $\log_2$(fold change)| ≥ 2 and adj. *P* ≤ 0.05.

(B) Whole-proteome profiling of SUDHL5 cells treated with 10 nM **TCIP3**, 250 nM dCBP1, 10 nM **NEG1**, or 10 nM **NEG2** for 6 h plotted with cutoffs of $\log_2$(fold change)| ≥ 2 and adj. *P* ≤ 0.05. For **(A)**, **(B)**: 3 biological replicates; *P*-values computed using a moderated t-test and adjusted by Benjamini-Hochberg.

(C) Quantification of individual peptides of interest from global proteomics of all treatments and timepoints matched across treatments. Lines represent median and interquartile range.

bioRxiv preprint doi: https://doi.org/10.1101/2025.03.14.643404; this version posted March 17, 2025. The copyright holder for this preprint (which was not certified by peer review) is the author/funder, who has granted bioRxiv a license to display the preprint in perpetuity. It is made available under aCC-BY 4.0 International license.

(D) Signaling pathways (MSigDB Hallmark 2020) enriched after 24 h treatment of indicated compounds in SUDHL5 in significantly decreased (adj. $P < 0.05$, $\log_2$(foldchange) < -1) or significantly increased (adj. $P < 0.05$, $\log_2$(foldchange) > 1) proteins.

bioRxiv preprint doi: https://doi.org/10.1101/2025.03.14.643404; this version posted March 17, 2025. The copyright holder for this preprint (which was not certified by peer review) is the author/funder, who has granted bioRxiv a license to display the preprint in perpetuity. It is made available under aCC-BY 4.0 International license.

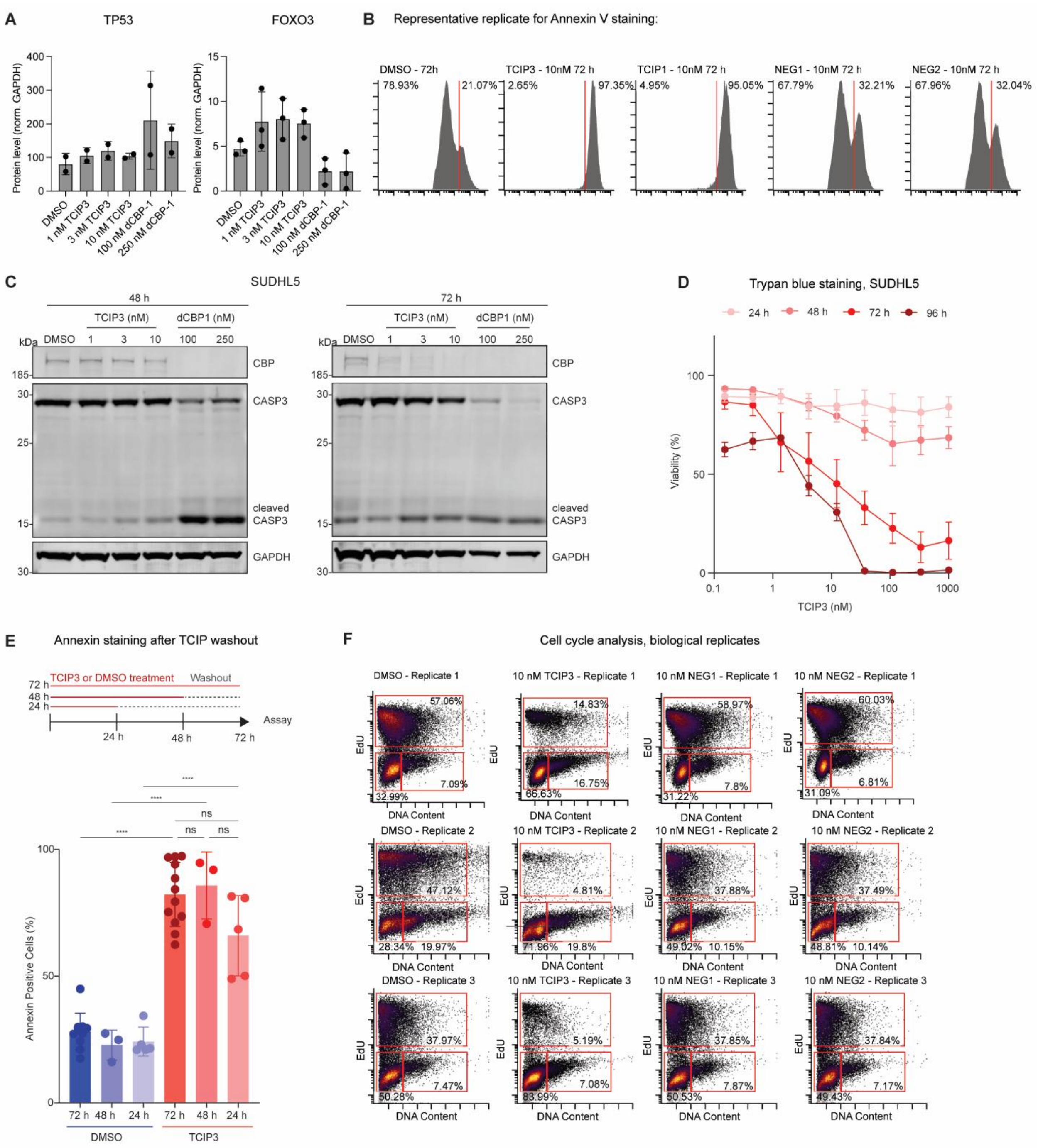

Supplemental Figure 6. Characterization of Apoptosis and Cell Cycle Arrest

(A) Quantification of TP53 and FOXO3 protein levels from Fig. 4D**;** *P*-values computed by Fisher's LSD test after ANOVA; only the comparisons to DMSO were computed; no comparisons were significant.

(B) Representative (of 3-12 biological replicates) Annexin V staining replicate of SUDHL5 cells treated with **TCIP3, NEG1**, **NEG2**, or DMSO for 72 h.

(C) Western blot of caspase 3 in SUDHL5 cells treated with indicated compounds and doses after 48 and 72 h; representative of 3 biological replicates**.**

bioRxiv preprint doi: https://doi.org/10.1101/2025.03.14.643404; this version posted March 17, 2025. The copyright holder for this preprint (which was not certified by peer review) is the author/funder, who has granted bioRxiv a license to display the preprint in perpetuity. It is made available under aCC-BY 4.0 International license.

(D) Percent of cells alive after Trypan blue staining (Trypan blue negative); SUDHL5 cells treated with **TCIP3** for indicated timepoints and doses. 3 biological replicates; mean ± sem.

(E) Quantification of Annexin V positive SUDHL5 cells treated with DMSO or 10 nM **TCIP3** for 24, 48, or 72 h and then washed with PBS and replaced with media for 48, 24, or 0 h, respectively; *P*-values adjusted by Tukey's test after analysis of variance (ANOVA); ****: adj. $P < 0.0001$; all unlabeled comparisons were not significant. 72 h data corresponds to Fig. 4F.

(F) Individual cell cycle analysis biological replicates corresponding to Fig. 4H.

bioRxiv preprint doi: https://doi.org/10.1101/2025.03.14.643404; this version posted March 17, 2025. The copyright holder for this preprint (which was not certified by peer review) is the author/funder, who has granted bioRxiv a license to display the preprint in perpetuity. It is made available under aCC-BY 4.0 International license.

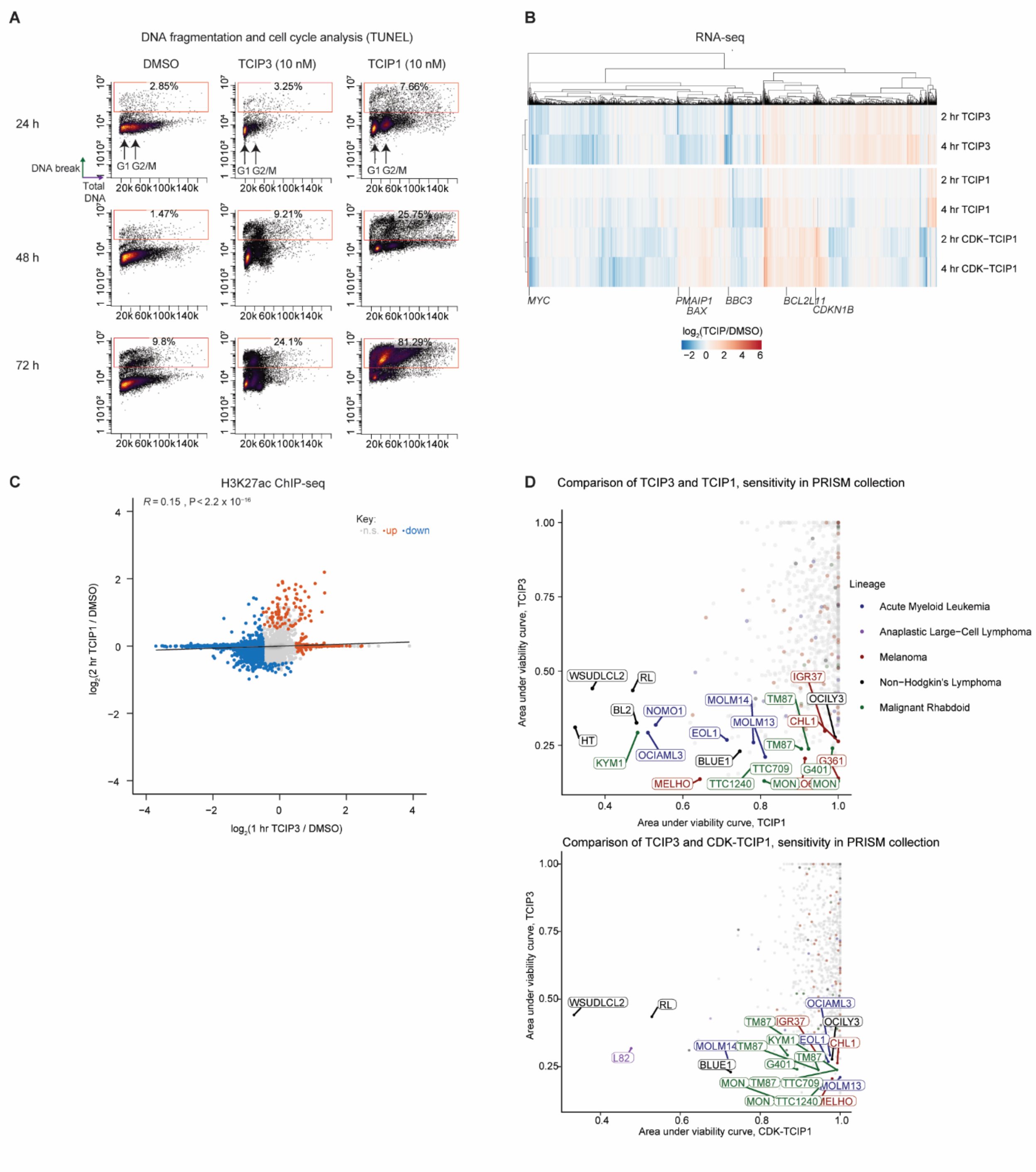

Supplemental Figure 7. Comparison of BCL6-targeting TCIPs
(A) Concurrent analysis of apoptosis and cell cycle effects of TCIPs by terminal deoxynucleotidyl transferase dUTP nick end labeling (TUNEL) and total DNA content co-staining of SUDHL5 cells treated with DMSO, 10 nM TCIP1, or 10 nM **TCIP3** for 24, 48, or 72 h; corresponds to Fig. 4G; representative of 4 biological replicates; cell cycle phases labeled.

bioRxiv preprint doi: https://doi.org/10.1101/2025.03.14.643404; this version posted March 17, 2025. The copyright holder for this preprint (which was not certified by peer review) is the author/funder, who has granted bioRxiv a license to display the preprint in perpetuity. It is made available under aCC-BY 4.0 International license.

(B) Unbiased clustering of differential gene expression caused by **TCIP3**, TCIP1[39], and CDK-TCIP1[40]; differential genes were defined by adj. $P \leq 0.05$ and $|\log_2(\text{fold change})| \geq 0.5$, *P*-values computed by two-sided Wald test and adjusted for multiple comparisons by Benjamini-Hochberg; 3-4 biological replicates.
(C) Correlation of changes at all H3K27ac peaks (86,087) between TCIP1 and **TCIP3** treatment; colored are peaks that change significantly for either treatment, significance defined by adj. $P \leq 0.05$ and $|\log_2(\text{fold change})| \geq 0.5$, *P*-values computed by two-sided Wald test and adjusted for multiple comparisons by Benjamini-Hochberg; 2 biological replicates; *R* computed by Pearson's correlation and *P*-value computed by two-sided Student's t-test.
(D) Comparison of sensitivity of cell line to **TCIP3** with sensitivity to TCIP1[39] and CDK-TCIP1[40] in ~900 cancer cell lines in PRISM[55,96]; labeled are top-ranked (most sensitive) lines colored by lineage.

bioRxiv preprint doi: https://doi.org/10.1101/2025.03.14.643404; this version posted March 17, 2025. The copyright holder for this preprint (which was not certified by peer review) is the author/funder, who has granted bioRxiv a license to display the preprint in perpetuity. It is made available under aCC-BY 4.0 International license.

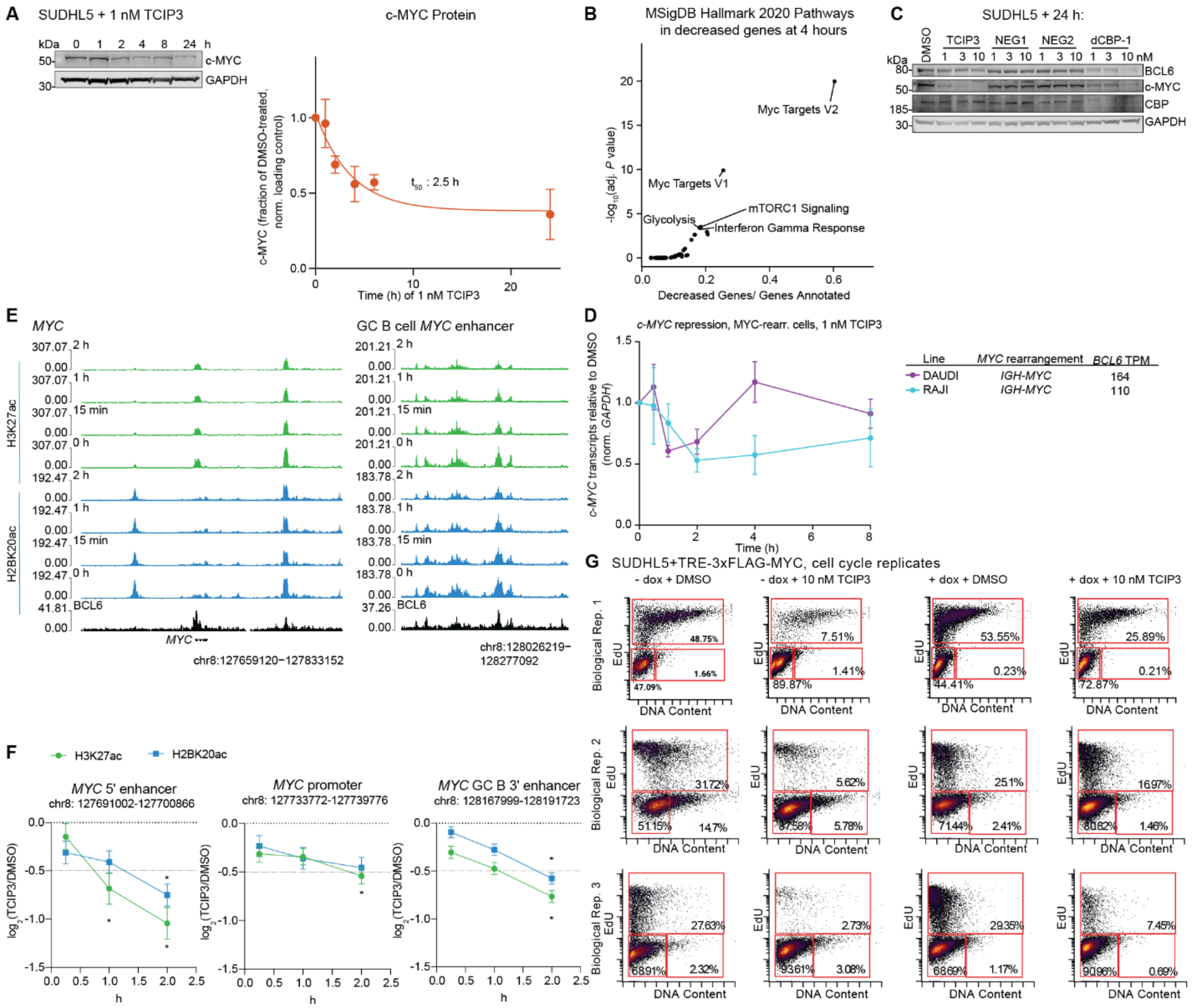

Supplemental Figure 8. Characterization of c-MYC Repression

(A) c-MYC protein in SUDHL5 cells treated with 1 nM **TCIP3** for indicated timepoints; blot representative of 3 biological replicates, plotted is c-MYC normalized to loading controls and DMSO levels, fit to a one-phase exponential decay model, mean ± s.e.m.

(B) Signaling pathways (MSigDB Hallmark 2020) significantly enriched among significantly decreased transcripts (adj. $P \leq 0.05$ and $\log_2$(fold change) ≤ -0.5) after 4 h treatment of **TCIP3** (1 nM) in SUDHL5 cells.

(C) Protein levels in SUDHL5 cells treated with indicated compounds for 24 h.

(D) *c-MYC* transcripts in Burkitt's lymphoma cells with chromosomal rearrangement of the *MYC* locus to the immunoglobulin heavy chain (IGH) locus[79,80]; *BCL6* expression from[95]; cells treated with 1 nM of **TCIP3** normalized to *GAPDH* and DMSO treatment as quantified through RT-qPCR; 3 biological replicates, mean ± s.e.m.

(E) Changes in H2BK20ac and H3K27ac with time at the promoter of *c-MYC* and its upstream and downstream enhancer regions; BCL6 track is CUT&RUN in untreated SUDHL5 cells, tracks merged from two biological replicates and sequence-depth normalized and, for histone acetylation ChIP-seq, also input-subtracted.

bioRxiv preprint doi: https://doi.org/10.1101/2025.03.14.643404; this version posted March 17, 2025. The copyright holder for this preprint (which was not certified by peer review) is the author/funder, who has granted bioRxiv a license to display the preprint in perpetuity. It is made available under aCC-BY 4.0 International license.

(F) Quantification of changes in H2BK20ac and H3K27ac differential peaks around the *c-MYC* locus; *: adj. $P \leq 0.05$ and $\log_2$(fold change) ≤ -0.5; 2 biological replicates, mean ± s.e.m.; *P*-values computed by two-sided Wald test and adjusted by multiple comparisons by Benjamini-Hochberg.

(G) Individual biological replicates of cell cycle analysis of SUDHL5$^{TRE\text{-}3xFLAG\text{-}MYC}$ cells treated with or without 1 μg/mL doxycycline dissolved in ethanol or vehicle 24 h prior to 24 h treatment with 10 nM **TCIP3** or DMSO.

bioRxiv preprint doi: https://doi.org/10.1101/2025.03.14.643404; this version posted March 17, 2025. The copyright holder for this preprint (which was not certified by peer review) is the author/funder, who has granted bioRxiv a license to display the preprint in perpetuity. It is made available under aCC-BY 4.0 International license.

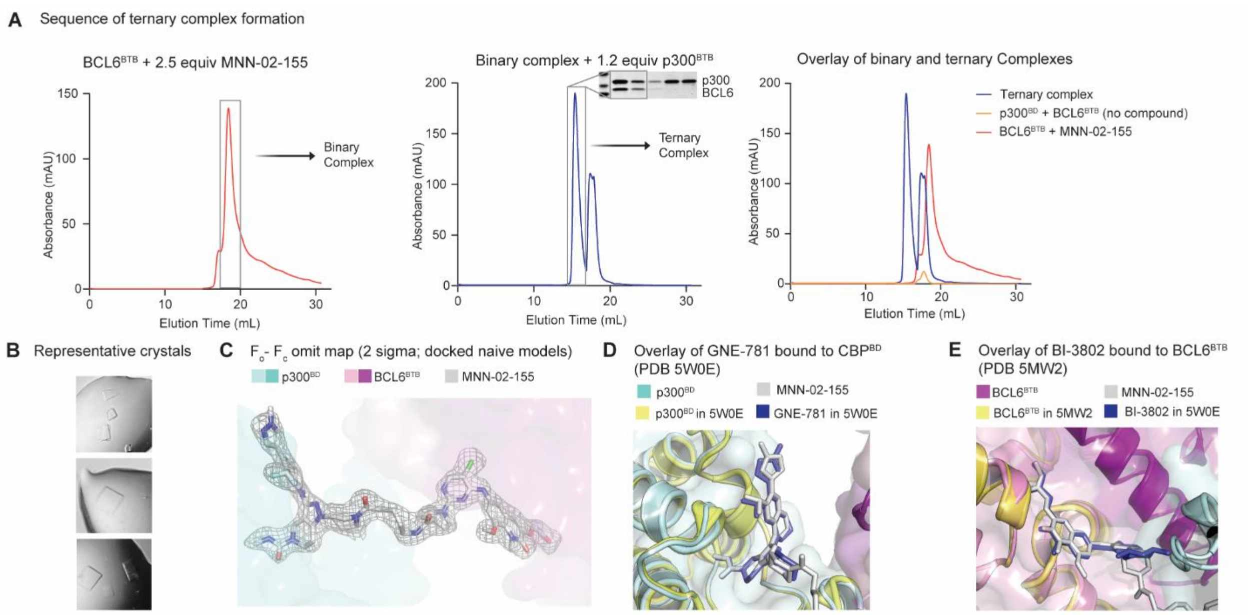

Supplemental Figure 9. Structural Analyses of KAT-TCIPs

(A) Schematic of biochemical formation of a ternary complex between **MNN-02-155**, P300^{BD}, and BCL6^{BTB} domains. Binary and ternary complexes were purified by size-exclusion and concentrated to desired concentrations.

(B) Representative images of crystals containing the ternary complex.

(C) F_o-F_c map of **MNN-02-155** in the co-crystal structure with p300^{BD} and BCL6^{BTB} domains.

(D) Overlay of the co-crystal structure with the published structure of GNE-781 bound to CBP (PDB: 5W0E).

(E) Overlay of the co-crystal structure with the published structure of BI-3812 bound to BCL6 (PDB: 5MW2).

bioRxiv preprint doi: https://doi.org/10.1101/2025.03.14.643404; this version posted March 17, 2025. The copyright holder for this preprint (which was not certified by peer review) is the author/funder, who has granted bioRxiv a license to display the preprint in perpetuity. It is made available under aCC-BY 4.0 International license.

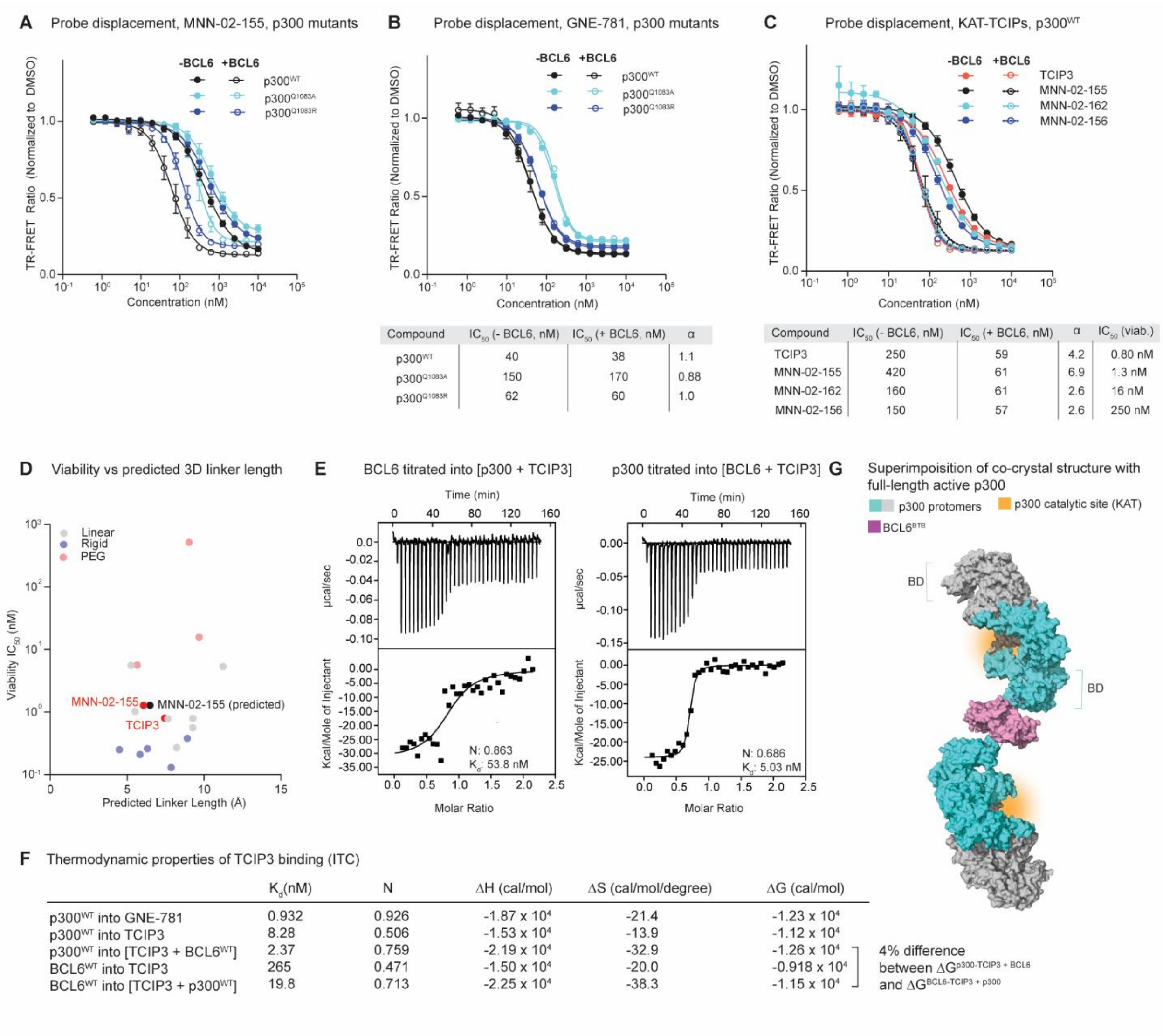

Compound	IC_{50} (- BCL6, nM)	IC_{50} (+ BCL6, nM)	α
$p300^{WT}$	40	38	1.1
$p300^{Q1083A}$	150	170	0.88
$p300^{Q1083R}$	62	60	1.0

Compound	IC_{50} (- BCL6, nM)	IC_{50} (+ BCL6, nM)	α	IC_{50} (viab.)
TCIP3	250	59	4.2	0.80 nM
MNN-02-155	420	61	6.9	1.3 nM
MNN-02-162	160	61	2.6	16 nM
MNN-02-156	150	57	2.6	250 nM

	K_d(nM)	N	ΔH (cal/mol)	ΔS (cal/mol/degree)	ΔG (cal/mol)
$p300^{WT}$ into GNE-781	0.932	0.926	-1.87×10^4	-21.4	-1.23×10^4
$p300^{WT}$ into TCIP3	8.28	0.506	-1.53×10^4	-13.9	-1.12×10^4
$p300^{WT}$ into [TCIP3 + $BCL6^{WT}$]	2.37	0.759	-2.19×10^4	-32.9	-1.26×10^4
$BCL6^{WT}$ into TCIP3	265	0.471	-1.50×10^4	-20.0	-0.918×10^4
$BCL6^{WT}$ into [TCIP3 + $p300^{WT}$]	19.8	0.713	-2.25×10^4	-38.3	-1.15×10^4

Supplemental Figure 10. Biophysical Analyses of KAT-TCIPs

(A) Binary TR-FRET displacement curves of **MNN-02-155** corresponding to Fig. 7D.

(B) Binary TR-FRET displacement assay and cooperativity analysis evaluating the inhibition of GNE-781 against $p300^{WT}$, $p300^{1083A}$, $p300^{1083R}$ in the absence or presence of BCL6 (>>> K_d). n = 2-3 biological replicates; mean ± s.e.m.

(C) Binary TR-FRET displacement assay and cooperativity analysis evaluating the inhibition of **TCIP3, MNN-02-155, MNN-02-162, or MNN-02-156** against $p300^{WT}$ in the absence or presence of BCL6 (>>> K_d); cell viability IC_{50} after 72 h treatment in SUDHL5 (Supplemental Fig. 1) labeled in table. n = 2-3 biological replicates; mean ± s.e.m.

(D) IC_{50}s (nM) of KAT-TCIPs synthesized from GNE-781 treated for 72 h in SUDHL5 (from Supplemental Fig. 1A, 2A) plotted against predicted lowest-energy-conformation linker lengths (average of the 2-3 lowest energy conformations).

(E) ITC traces of binding events to form a ternary complex, including $BCL6^{BTB}$ (10 µM) titrated into $p300^{BD}$ (20 µM) and **TCIP3** (1 µM), or $p300^{BD}$ (20 µM) titrated into $BCL6^{BTB}$ (40 µM) and **TCIP3** (2 µM), and respective N and K_d values; corresponds to Fig. 6E; representative of 3 biological replicates.

bioRxiv preprint doi: https://doi.org/10.1101/2025.03.14.643404; this version posted March 17, 2025. The copyright holder for this preprint (which was not certified by peer review) is the author/funder, who has granted bioRxiv a license to display the preprint in perpetuity. It is made available under aCC-BY 4.0 International license.

(F) Thermodynamic parameters of binding measured from ITC. All measurements represent the mean of 2-3 biological replicates.

(G) Superimposition of the co-crystal structure of **MNN-02-155** engaged with $p300^{BD}$ and $BCL6^{BTB}$ with the active p300 core, containing two p300 protomers, one teal and one gray (PDB: 6GYR).

bioRxiv preprint doi: https://doi.org/10.1101/2025.03.14.643404; this version posted March 17, 2025. The copyright holder for this preprint (which was not certified by peer review) is the author/funder, who has granted bioRxiv a license to display the preprint in perpetuity. It is made available under aCC-BY 4.0 International license.

Supplemental Figure 11. Full Scans of Western Blots.

Supplemental Figure 12. Flow Gating Strategies.

Supplemental Table 1. Enriched proteins after p300 IP-MS in SUDHL5 cells, FLAG IP-MS in FLAG-tagged *BCL6* SUDHL5 cells, or after global proteome profiling after TCIP3, dCBP-1, NEG1, and NEG2 treatment in SUDHL5 cells.

Supplemental Table 2. Full results of enrichment of histone acetylation peaks in public transcription factor ChIP-seq datasets in blood-lineage cells (ChIP-atlas). Contains all significantly enriched TFs in H3K27ac and H2BK20ac differential peaks. Legend is on Sheet 1 of the Table.

Supplemental Table 3: Crystallographic data collection and refinement statistics.

Supplemental File 1: Chemical Synthesis and Characterization.

Supplemental Video 1: Co-Crystal Structure of MNN-02-155 in complex with p300^{BD} and BCL6^{BTB}.
